# Case Reports of Bacillus Calmette-Guérin (BCG) Osteomyelitis: A Rare Complication in Immunocompetent Infants

**DOI:** 10.7759/cureus.12410

**Published:** 2020-12-31

**Authors:** Yousra Ghoweba, Lemis Yavuz, Wafaa Faysal, Sattar Alshryda, Walid Abuhammour

**Affiliations:** 1 Pediatrics, Al Jalila Children's Hospital, Dubai, ARE; 2 Pediatrics, Dr. Sulaiman Al Habib Hospital, Dubai, ARE; 3 Pediatric Orthopedics, Al Jalila Children Specialty Hospital, Dubai, ARE; 4 Infectious Diseases, Al Jalila Children's Speciality Hospital, Dubai, ARE

**Keywords:** case reports, bcg, vaccination, osteomyelitis, rare, complication, immunocompetent, infants

## Abstract

Bacillus Calmette-Guérin (BCG) vaccine is a live attenuated vaccine used globally since 1921, and in the United Arab Emirates (UAE), it is administered to all newborns within the first few days of life for well-established benefits. BCG osteomyelitis is a rare complication that should be considered while assessing osteomyelitis in children. This report describes three cases of BCG osteomyelitis involving proximal metaphysis of the humerus in 11 months and three months old immunocompetent male infants and the left proximal tibia in a two-year-old immunocompetent female. To the best of our knowledge, these are the first cases to be reported in the UAE. The report outlines in detail how to make a timely diagnosis by explaining the insidious clinical presentation of BCG osteomyelitis, including its radiologic, microbiologic, and histologic aspects. As well, it outlines the treatment course carried out for these three patients. As such, this report will aid physicians in staying vigilant for such rare complication and commencing early treatment.

## Introduction

Bacillus Calmette-Guérin (BCG), a live attenuated vaccine derived from Mycobacterium bovis, was first introduced in 1921 in Lille, France [1]. Its effectiveness led to worldwide usage in the first few days of life, especially in areas with a high prevalence of tuberculosis (TB). It plays an important role in preventing serious childhood TB infections like meningeal and miliary TB [2]. Mild side effects following BCG vaccination like lymphadenitis, abscess, local swelling can occur in 3.3% of the population in the first year of life [1], while serious side effects like osteomyelitis remain very rare. Thus, when three cases at the age of 11 months, three months, and two-year-old were encountered within a span of a year at the same hospital, it triggered the need for writing a case report. The main objective of the report is to bring awareness to the condition and aid physicians in making a timely diagnosis of BCG osteomyelitis and commencing the appropriate treatment.

## Case presentation

Case 1

History

An 11-month-old male was referred to our hospital with right arm pain and decreased range of motion for one week. There was no history of trauma, fever, or constitutional symptoms. The child had a chest infection when he was nine months old, but otherwise, he was a healthy child. There was no past medical history of pulmonary TB or contact with TB patients. The patient had an up to date immunization records, including the BCG vaccine at birth.

Examination

The child appeared well, afebrile, and reluctant to move his right arm. No joint swelling, erythema, warmth, tenderness, or deformity were noted. 

Blood Tests

Normal white blood cells count (WBC; 8.43 x 103/mcL) and C-reactive protein (CRP; 1.14 mg/L), but an elevated erythrocyte sedimentation rate (ESR; 28 mm/hr) and microcytic hypochromic anemia; hemoglobin (Hb; 9.9 g/dl), mean corpuscular volume (MCV; 64 fL) and mean corpuscular hemoglobin concentration (MCHC; 30.3 g/dl) were noted. Blood culture was negative.

Imaging

**Figure 1:**
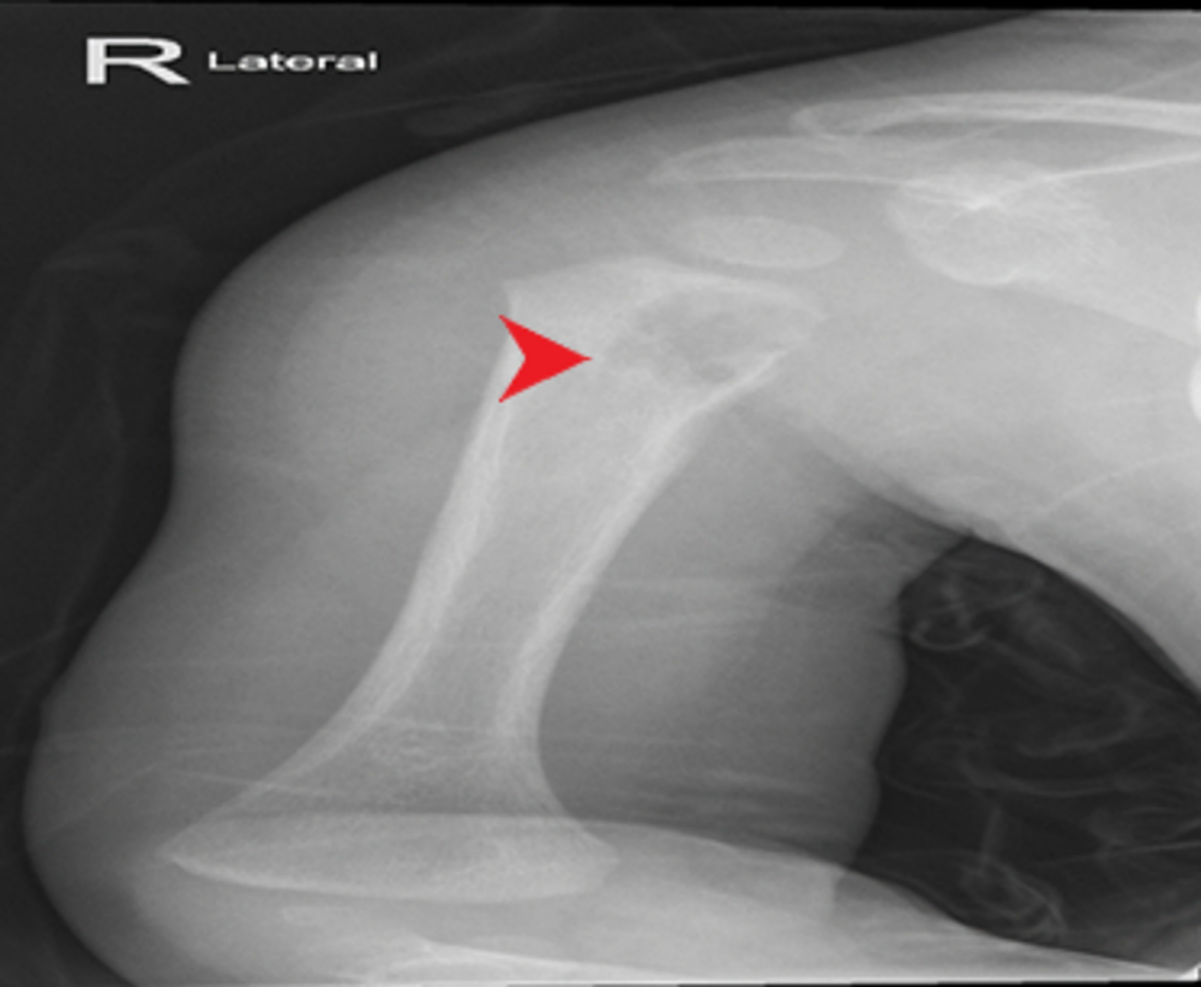
X-ray shows a sharply demarcated lytic lesion with cortical erosion in the right proximal humerus.

**Figure 2:**
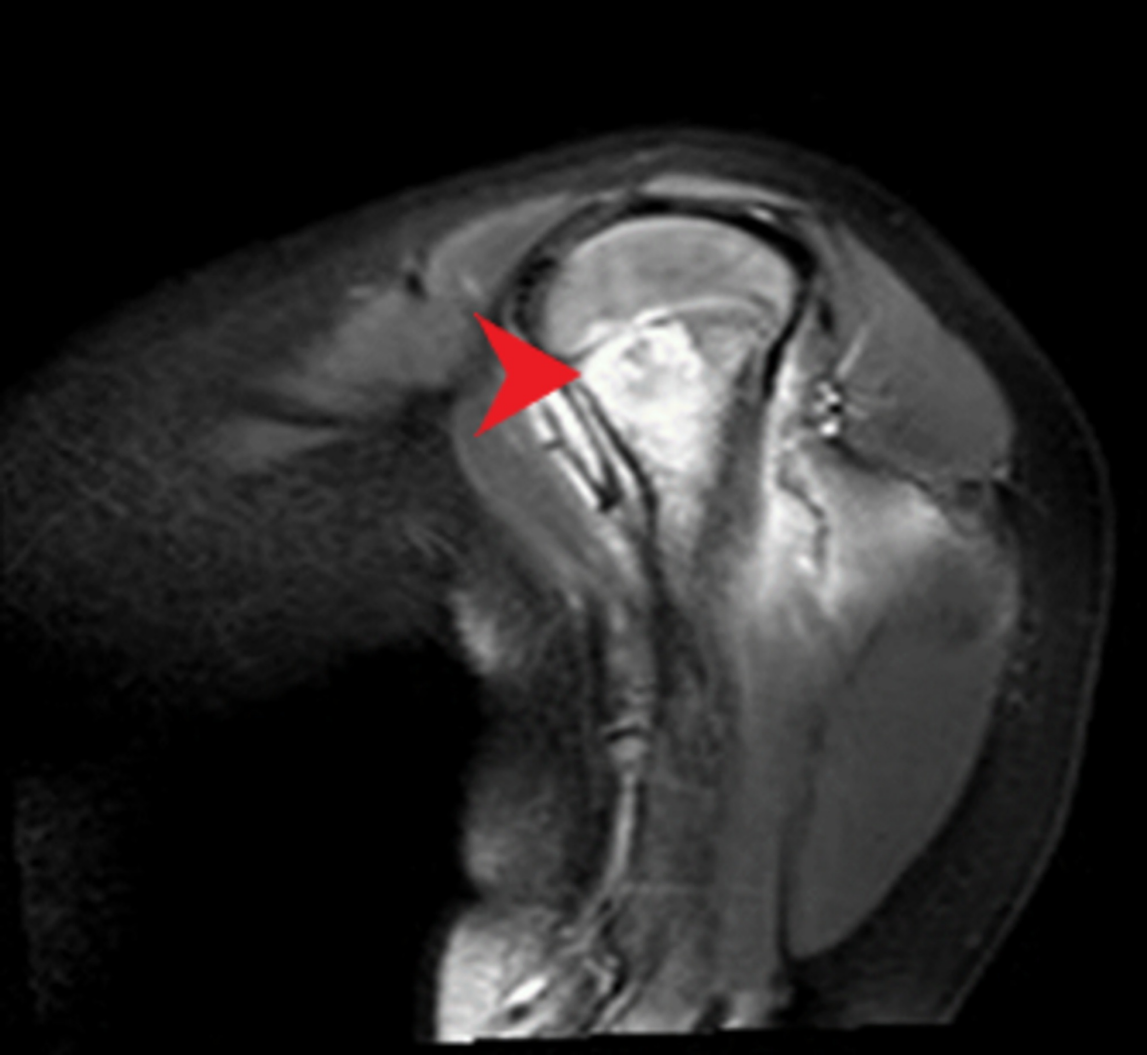
MRI of the proximal humerus showing high signal of the bone and adjacent soft tissues.

Right arm plain radiograph (Figure 1) and magnetic resonance imaging (Figure 2) showed an eccentric osteolytic lesion in the proximal metaphysis of the right humerus, flagging the possibility of osteomyelitis or even a tumor. Chest plain radiograph was negative for any tuberculous foci or consolidation.

Further Investigations

**Figure 3:**
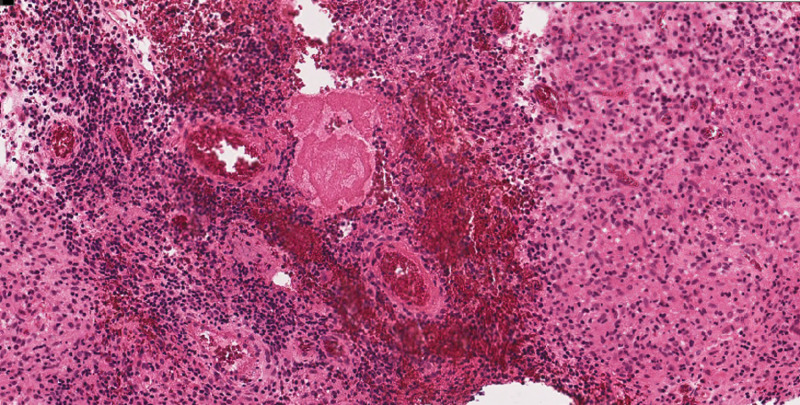
Histopathology shows epithelioid histiocytic proliferation with multinucleated giant cells, consistent with granulomatous osteomyelitis.

A purified protein derivative (PPD) skin test showed a 10 mm induration (positive) at 48 and 72 hours. However, the QuantiFERON® test was negative. Bone biopsy was performed, and the sample was sent for gram stain, culture, acid-fast bacilli stain, Mycobacterium culture, and histopathology. Gram stain, culture, and acid-fast bacilli stain were all negative. The Mycobacterium culture grew Mycobacteria tuberculosis complex resistant to pyrazinamide after three weeks incubation, resembling Mycobacterium bovis secondary to BCG. Bone histopathology showed caseating granulomatous inflammatory tissue (Figure 3) consistent with tuberculosis osteomyelitis. Moreover, full immunologic workup including immunoglobulins, pneumococcal antibodies, tetanus/diphtheria antibodies, neutrophil oxidative burst assay, and mendelian susceptibility to Mycobacteria genetic study were all normal. 

Case 2

History

A three-month-old male with an insignificant past medical history presented with pain upon moving his left arm. There was no history of trauma, fever, or any constitutional symptoms. There was no past medical history of pulmonary TB or contact with TB patients. His immunization records were up to date, including the BCG vaccine at birth.

Examination

The child was holding his left arm in an adducted position with a decreased range of motion and tenderness on palpation.

Blood Tests

Mildly elevated WBC (16 x 103/mcL) and ESR (12mm/hr), but a normal CRP (2.87 mg/L) with normocytic normochromic anemia; Hb (10.2g/dl), MCV (81.2 fL) and MCHC (32.4 g/dl) were noted. Blood culture was negative.

Imaging

**Figure 4:**
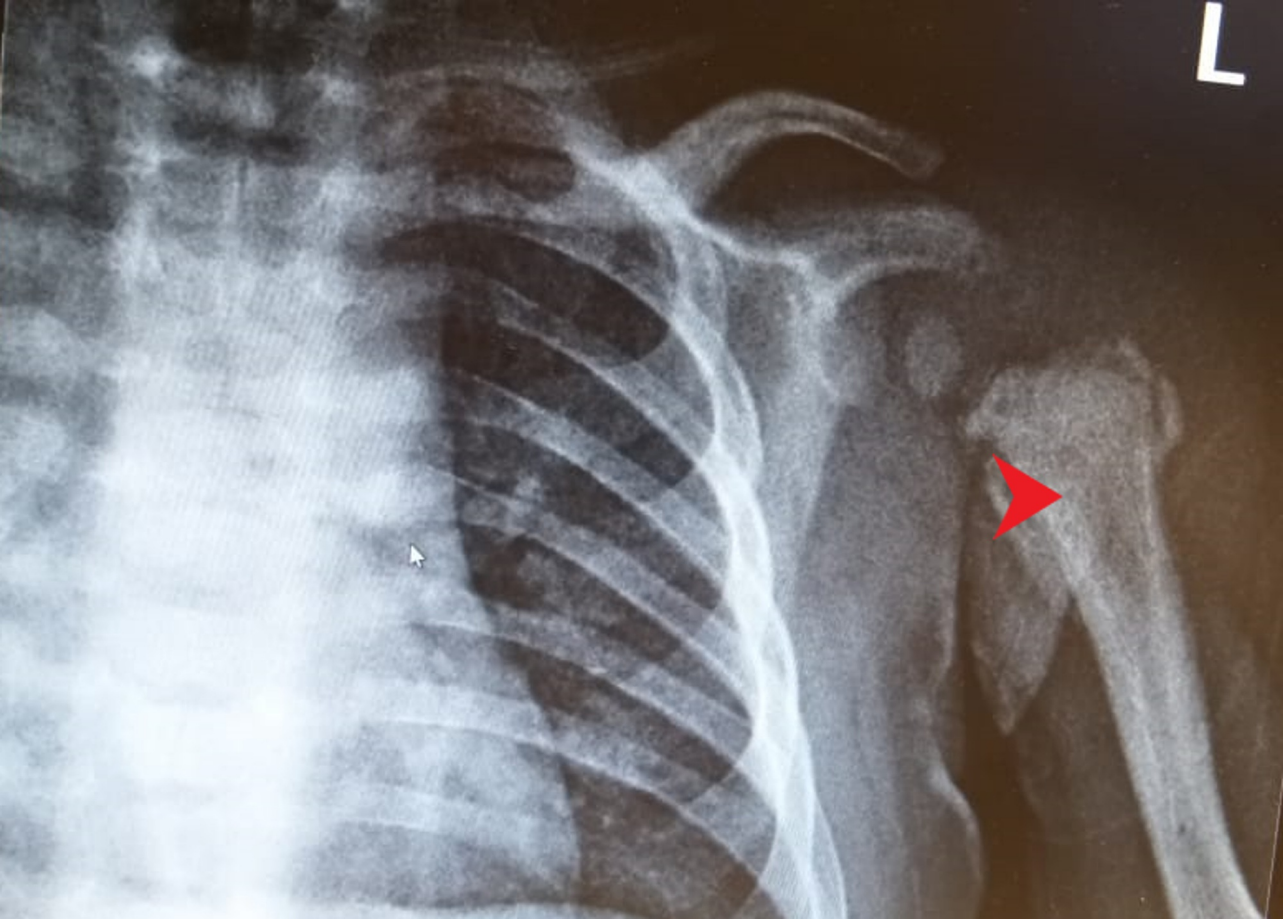
X-ray of the left shoulder shows periosteal reaction with new bone formation.

**Figure 5:**
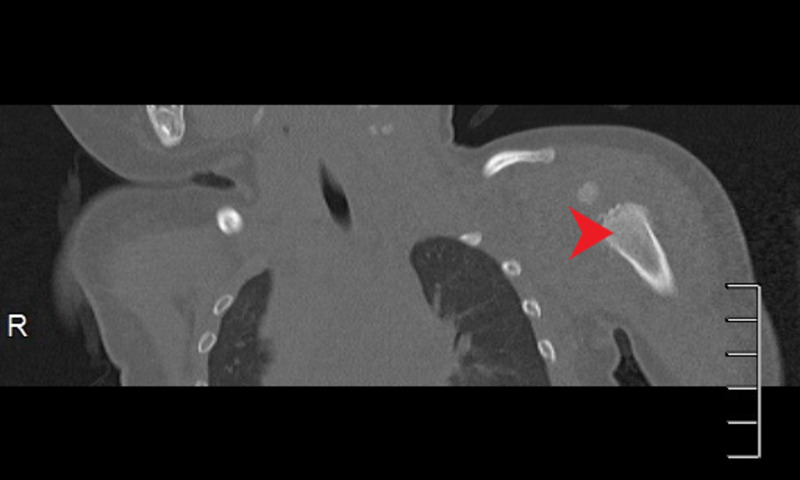
CT scan showing periosteal reaction in metaphysis of left humerus.

Plain radiograph (Figure 4), computerized tomography (Figure 5), and MRI scans of the left arm showed a periosteal reaction in the bone with new bone formation at the metaphysis.

Further Investigations

PPD skin test showed an 11 mm induration (positive) at 48 and 72 hours. Bone Mycobacterium culture grew Mycobacteria tuberculosis complex resistant to pyrazinamide after three weeks incubation, resembling Mycobacterium bovis secondary to BCG. The histopathology showed caseating granulomatous inflammation. Full immunologic workup including immunoglobulins, pneumococcal antibodies, tetanus/diphtheria antibodies, neutrophil oxidative burst assay, and mendelian susceptibility to Mycobacteria genetic study were all normal. 

Case 3

*History*:

A two-year-old female was referred to our hospital with left knee pain and swelling for two months. There was no history of trauma, fever, or weight loss; however, night sweats were reported. There was no past medical history of pulmonary TB or contact with TB patients. The patient had an up to date immunization records, including the BCG vaccine at birth.

Examination

The child appeared well, afebrile with swelling and tenderness of his left proximal tibia. No overlying erythema, warmth, deformity, or difficulty bearing weight were noted.

Blood Tests

Normal WBC (7.88 x 103/mcL) and CRP (1.16 mg/L), but an elevated ESR (29mm/hr) were noted as well as normal Hb (12.5 g/dl), MCV (76.2 fL) and MCHC (33.1g/dl). Blood culture was negative.

Imaging

**Figure 6:**
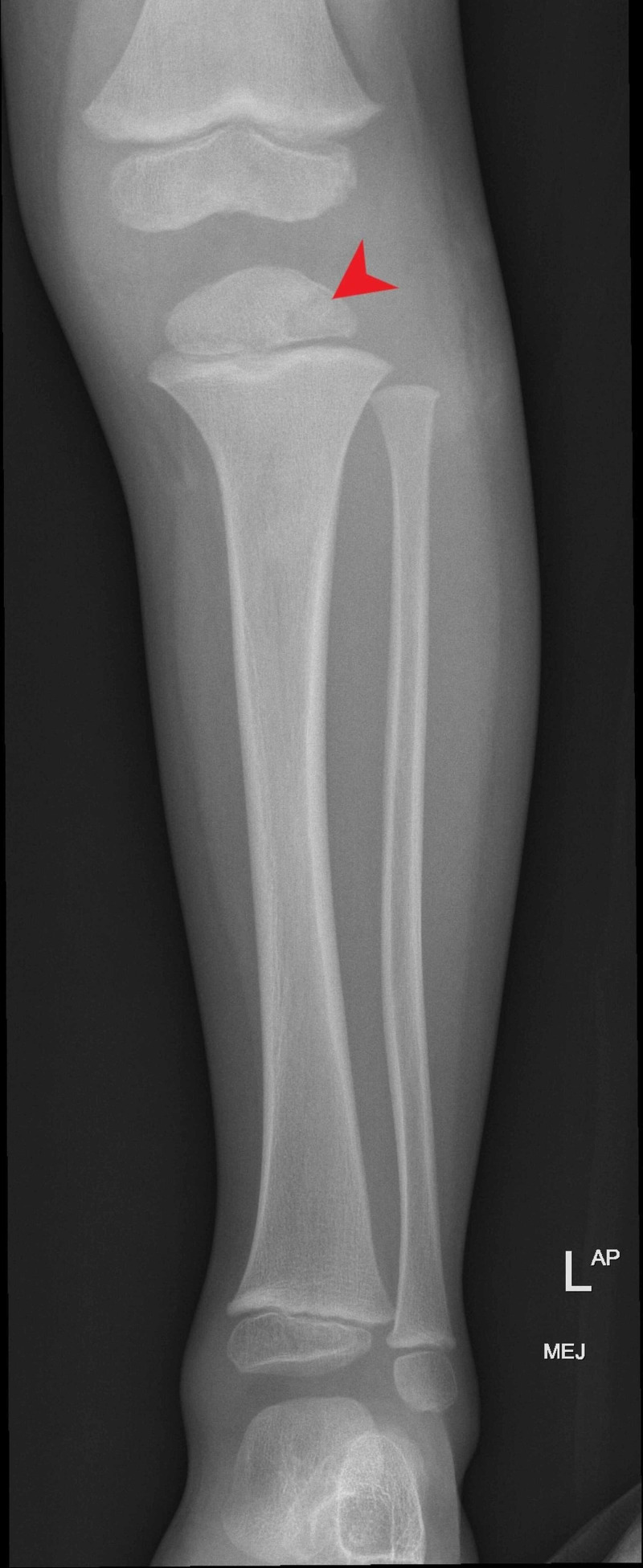
X-ray shows osteolytic lesions of the left proximal tibia.

Tibia plain radiograph (Figure 6) showed osteolytic lesions in the proximal metaphysis of the left tibia.

Further Investigations

The QuantiFERON® test was positive. Bone biopsy was performed, and the sample was sent for gram stain, culture, acid-fast bacilli stain, Mycobacterium culture, and histopathology. Gram stain and culture were negative; however, acid-fast bacilli came back positive. The Mycobacterium culture grew Mycobacteria tuberculosis complex resistant to pyrazinamide after three weeks incubation, resembling Mycobacterium bovis secondary to BCG. Bone histopathology showed caseating granulomatous inflammatory tissue consistent with tuberculosis osteomyelitis. Moreover, full immunologic workup including immunoglobulins, pneumococcal antibodies, tetanus/diphtheria antibodies, neutrophil oxidative burst assay, and mendelian susceptibility to mycobacteria genetic study were all normal. 

Diagnosis and treatment

The provisional list of diagnoses included subacute osteomyelitis, tuberculosis osteomyelitis, Ewing's sarcoma, and Langerhans's histiocytosis. However, the histological findings of caseating granulomatous inflammation in our cases narrowed the potential diagnoses to BCG osteomyelitis, tuberculosis osteomyelitis, or sarcoidosis. However, the subsequent positive Mycobacterium culture confirmed the diagnosis of BCG osteomyelitis.

**Figure 7:**
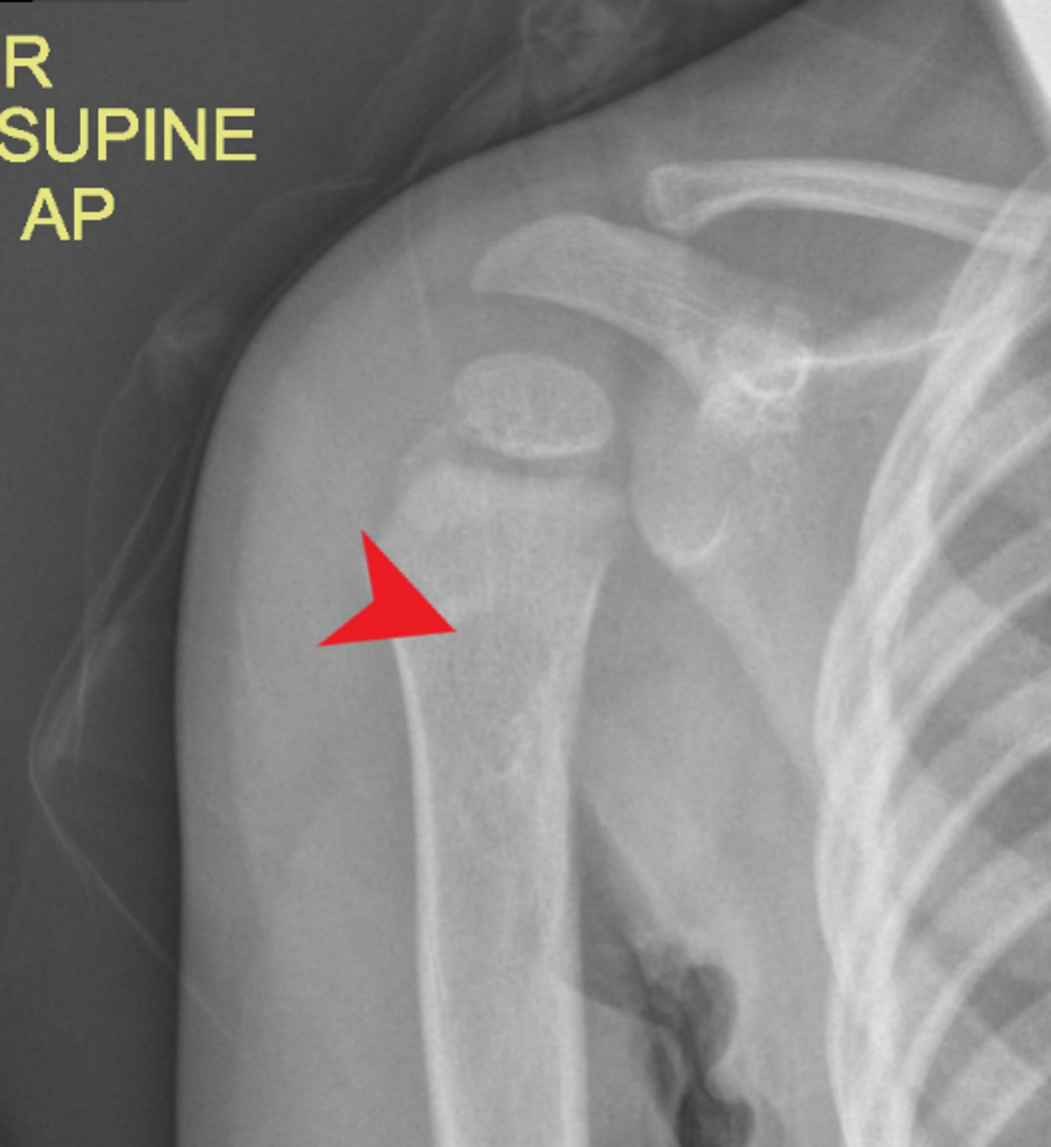
X-ray shows healing of the osteolytic lesion without any periosteal reaction detected.

Our first two patients were initially put on clindamycin after the imaging results. They were then put on anti-tuberculosis treatment after histopathology showed granulomatous inflammation, although the Mycobacterium culture results were not available yet. The triple anti-tuberculosis therapy, including isoniazid, rifampin, and ethambutol, was commenced for a 12 months period in our three cases. Patients showed clinical improvement on the anti-tuberculosis treatment. Our first case showed healing of the radiolucent area without any periosteal reaction detected on repeat X-ray (Figure 7) after completing the treatment. X-ray is yet to be repeated for our second and third cases after completing 12 months course of treatment.

## Discussion

BCG osteomyelitis is a rare complication following BCG vaccination. It occurs in about one in 80,000 to 100,000 children who received BCG vaccination [1-3]. This complication is often undiagnosed and misdiagnosed by healthcare professionals due to diagnostic difficulties and lack of awareness. It was reported in one study conducted in Taiwan involving 38 patients with BCG osteomyelitis that the average time from the first presentation to final management was between 1.6 to 2.1 months [4], which is attributed to late diagnosis.

Although BCG osteomyelitis is a serious complication, it tends to follow an indolent clinical course over weeks to months. Patients are usually well-appearing, with minimal symptoms. Mild arm pain on movement with mild tenderness and preference to use the unaffected limb was reported in our patients. The study conducted in Taiwan reported that 66% of the cases presented with a mass, 58% with tenderness, and 50% with limping if lower limp was involved [4].

It can occur at the site of vaccine injection (the left upper arm as per the standard) as seen in our second case or at a distant site as in our first and third patient. The latter may indicate hematogenous dissemination of Mycobacterium bovis [5-6]. It also commonly affects extremities more than axial bones [1, 4], and around 80% occur at metaphysis or epiphysis of long bones when extremities are affected [4], as supported by our findings.

Laboratory findings are usually insignificant with normal WBC and CRP. ESR can be slightly elevated, as is the case with our patients. This aids in differentiating BCG osteomyelitis from other possible differential diagnoses like acute streptococcal or staphylococcal osteomyelitis [7].

Plain radiographs typically show a well-demarcated osteolytic lesion with periosteal reaction. MRI scan usually shows a destructive and patchy lesion [5]. These make clinicians suspicious of Ewing's sarcoma, eosinophilic granuloma, or Langerhans’s histiocytosis [3, 5, 8].

Bone tissue biopsy and histopathologic evaluation are extremely valuable in reaching a definitive diagnosis. Histopathology typically shows granulomatous inflammation with macrophages and necrosis, which is pathognomonic for both tuberculous and BCG osteomyelitis. However, in BCG osteomyelitis, acid-fast bacilli stain is usually negative, whereas Mycobacterium bovis culture is positive. Mycobacterium bovis is intrinsically resistant to pyrazinamide, and this could be an early hint towards Mycobacterium bovis - BCG strain infection [9]. The latter usually takes weeks to grow, as was the case in our patients. Several reports highlighted the importance of PCR as a rapid, sensitive, and specific tool to diagnose Mycobacterium bovis infection [5]. PCR is considered a useful tool because of its ability to rapidly detect Mycobacterium tuberculosis complex before tissue culture results are available. This aids in early diagnosis and commencement of early treatment. Early management provides a better clinical outcome for patients and helps prevent future complications of growth plate destruction that can affect growth and impair ambulation [3].

In another study from Taiwan, eight out of 15 patients (53%) had a positive tuberculin purified protein derivative (PPD) test, and multiple case reports showed that PPD is usually positive while QuantiFERON test tends to be negative [10], as seen in case 1 and 2.

Another vital point to consider is the immunologic status of the patient. We did a full immunologic workup including immunoglobulins, antibodies to vaccines, neutrophil oxidative burst assay, and mendelian susceptibility to mycobacteria genetic study to assess if the patient is immunodeficient and at risk of mycobacterial infections.

The incubation period of BCG osteomyelitis is around six months; however, many authors reported that it could occur from three months to five years [5, 11], as in our second case, which occurred at the age of three months. Hence, clinicians should always keep BCG osteomyelitis in mind if a patient presents with a subacute clinical course, histologic evidence of granulomatous inflammation, and immunization record of BCG vaccine even if the age is below six months.

Our patients were put on anti-tuberculosis treatment after the histopathology showed granulomatous inflammation, although the Mycobacterium culture results were not available yet. This signifies the importance of having a high clinical suspicion and linking clinical presentation along with diagnostic clues to reach the right diagnosis even in the absence of bacterial isolation. The anti-tuberculosis triple therapy included isoniazid, ethambutol, and rifampin. A literature review showed no consensus on a management protocol for BCG osteomyelitis, and that surgical debridement is controversial. The optimal treatment duration is not well established yet; however, in most cases, continued treatment for six to 12 months [9]. A retrospective analysis of 222 cases of BCG osteomyelitis suggested using triple therapy of isoniazid, ethionamide, and streptomycin for one month, then isoniazid with ethionamide or rifampin for four months, followed by isoniazid monotherapy for 12 months [11]. We decided to treat our patients with triple therapy for 12 months. All of our patients showed clinical improvement as well as a radiographic resolution of the osteolytic lesion, as seen in case 1.

## Conclusions

BCG osteomyelitis should always be kept in mind when a child presents with an insidious clinical course of limited movement of the affected limb even without a history of pulmonary TB or contact with TB patient. Bone biopsy is recommended to confirm the diagnosis by histopathology to commence early treatment.

## References

[1] Gharehdaghi M, Hassani M, Ghodsi E, Khooei A, Moayedpour A (2015). Bacille Calmette-Guérin osteomyelitis. Arch Bone Jt Surg.

[2] Chan PK, Ng BK, Wong CY (2010). Bacille Calmette-Guérin osteomyelitis of the proximal femur. Hong Kong Med J.

[3] Foucard T, Hjelmstedt A (1971). BCG-osteomyelitis and -osteoarthritis as a complication following BCG-vaccination. Acta Orthop Scand.

[4] Chiu NC, Lin MC, Lin WL (2015). Mycobacterium bovis BCG-associated osteomyelitis/osteitis, Taiwan. Emerg Infect Dis.

[5] Al-Jassir FF, Aldeeri RA, Alsiddiky AM, Zamzam MM (2012). Osteomyelitis following Bacille Calmette-Guerin vaccination. Saudi Med J.

[6] Selvestravičius R, Sučilienė E, Saniukas K, Bobelytė O, Usonis V (2016). Sternal osteomyelitis after Bacillus Calmette-Guérin vaccination. Pediatr Rep.

[7] Peltola H, Salmi I, Vahvanen V, Ahlqvist J (1984). BCG vaccination as a cause of osteomyelitis and subcutaneous abscess. Arch Dis Child.

[8] Bergdahl S, Felländer M, Robertson B (1976). BCG osteomyelitis: experience in the Stockholm region over the years 1961-1974. J Bone Joint Surg Br.

[9] Khan S, Stimec J, Kitai I (2015). Nonresponding osteomyelitis in a two-year-old boy. CMAJ.

[10] Spyridou C, Ragab H, Murphy RA (2019). Two cases of BCG osteomyelitis diagnosed through polymerase chain reaction/electrospray ionization-mass spectrometry technology. Clin Infect Dis.

[11] Kröger L, Korppi M, Brander E (1995). Osteitis caused by bacille Calmette-Guérin vaccination: a retrospective analysis of 222 cases. J Infect Dis.

